# Endoscopic Dilatation of Dominant Strictures in Primary Sclerosing Cholangitis

**DOI:** 10.1155/1998/41362

**Published:** 1998-08

**Authors:** Eric R. Lemmer, Phillipus C. Bornman

**Affiliations:** 1 MRC/UCT Liver Research Centre Groote Schuur Hospital Observatory Cape 7925 South Africa; 2 Surgical Gastroenterology E23 Groote Schuur Hospital Observatory Cape 7925 South Africa


### Endoscopic Dilatation of Dominant Strictures in Primary Sclerosing Cholangitis

### ABSTRACT

*Wagner, S.,, Gebel, M.,, Meier, P.,, Trautwein, C.,, Bleck, J.,, Nashan, B.,* and *Manns, M. P., (1996) Endoscopic Management of Biliary Tract Strictures in Primary Sclerosing Cholangitis. Endoscopy; 28: 546–551.*

***Background and Study Aims:*** **In a subgroup of patients, primary sclerosing cholangitis (PSC) is complicated by high-grade focal strictures of the bile ducts, and this can have an unfavourable influence on the natural course of the disease. The aim of this study was to evaluate the efficacy and safety of endoscopic treatment in this selected patient group.**
***Patients and Methods:*** **Twelve symptomatic patients with primary sclerosing cholangitis and major ductal strictures were included in a prospective study of endoscopic treatment. All patients were managed by repeated angioplasty-type balloon dilation and nasobiliary catheter perfusion. A minimum of two treatment sessions was used, and therapy was continued until satisfactory reopening of the strictures was obtained. Routine endoscopic follow-up was performed after three, six, 12, 18, and 24 months, and then at yearly intervals. The efficacy of therapy was assessed by evaluating clinical symptoms, laboratory data, and cholangiograms.**
***Results:*** **The long-term follow-up averaged 23 months (range: 12–50 months). Two to nine (mean:-three) treatment sessions were required to obtain satisfactory reopening of major biliary strictures. Eight patients showed considerable and sustained improvement. The mean serum bilirubin, alkaline phosphatase, $\gamma$-glutamyl-transpeptidase, and alanine aminotransferase levels fell significantly by 73% ($P$= 0.0164), 46% ($P$= 0.0022), 55% ($P$=0.0022), and 58% ($P$=0.0022), respectively. The average radiographic stricture score before treatment was 3.2 ±0.7 (mean ±SD), and after treatment decreased to 1.9 ± 0.8 ($P$= 0.0033). Three patients required liver transplantation seven, 12, and 40 months after the initiation of endoscopic treatment, due to a deterioration in hepatic function or an inability to exclude complex biliary malignancy. No major procedure-related side effects were observed.**
***Conclusions:*** **Our results suggest that the endoscopic treatment of PSC patients with dominant bile duct strictures is effective, safe, and well-tolerated. However, it is important not to overlook the potential development of cholangiocarcinoma.**

*Keywords:* Primary sclerosing cholangitis, bile duct strictures, ERCP dilatation

### PAPER DISCUSSION

The paper by Wagner *et al.*, reviewed the results of endoscopic treatment in twelve symptomatic patients with primary sclerosing cholangitis (PSC) complicated by dominant strictures of the extrahepatic bile ducts. Combining nasobiliary catheter perfusion and repeated balloon dilations, they demonstrated improvement in clinical, biochemical, and radiological parameters during a mean follow-up period of 23 months (range 12–50 months). Importantly, endoscopic therapy resulted in a significant drop in serum bilirubin levels, and septic complications following ERCP were minimal. Three patients required liver transplantation for progression to endstage liver failure or suspected complicating cholangiocarcinoma.

Although PSC usually affects the biliary tree diffusely, some patients have high-grade strictures of the extrahepatic biliary tree, often near or at the hepatic duct bifurcation [1], and where the cystic duct enters the bile duct. The term "dominant stricture" is imprecise, and may include any "flow limiting" stricture involving the extrahepatic biliary tree, even though it might not necessarily be the most severe stricture [2]. Some authors have recommended resection and/or bypass of the hepatic duct bifurcation for selected patients with advanced PSC and a dominant stricture who have developed persistent jaundice but who do not have cirrhosis [2, 3]. However, major surgery may compromise future transplant and the risk for the development of cholangiocarcinoma remains

[4, 5]. Furthermore, the presence of a dominant stricture on cholangiography has not been shown to be an independant predictor of survival in the various prognostic models that have been developed for PSC using multivariate analysis [6]. It is now generally accepted that patients with advanced PSC should proceed directly to transplant and that palliative surgical operations should be avoided.

Should endoscopic therapy be recommended for patients with advanced PSC and dominant strictures of the extrahepatic bile ducts? This form of treatment is theoretically appealing in PSC as it is much less invasive than biliary surgery, the biliary anatomy is not interfered with, treatments may be repeated, and biliary brushings can be taken for cytology. The results after endoscopic therapy would only need to be as good as (and not better than) those of surgical drainage/bypass operations for this to become the preferred mode of therapy for PSC and dominant strictures. However, the caveats for surgical and endoscopic therapy are essentially the same. In this study, half of the patients were cirrhotic. Biopsy was only performed "if indicated", so this figure may be an underestimate of the actual number of cirrhotic patients. We would caution against any biliary manipulations in PSC patients with endstage biliary cirrhosis, as the injection of contrast into a poorly drained biliary system may set up a vicious cycle of biliary sepsis and further interventional procedures, and the patient may forego the chance of a transplant due to uncontrolled sepsis. The endoscopic therapy given was also not minimally invasive: following initial ERCP, all patients in the study received saline perfusion of the biliary tree via a nasobiliary tube for a week, and up to nine (average three) ERCP sessions were required to achieve a satisfactory cholangiographic result. Nevertheless, nasobiliary perfusion may be important, and might have been a reason for the low incidence of biliary sepsis noted in this study.

It is particularly difficult to study the effect of endoscopic treatment, or for that matter any therapy, on the course PSC for a number of reasons: (1) PSC is uncommon (but not rare, as previously thought); (2) its course is variable albeit slowly progressive over many years; and (3) there is the unpredictable occurrence of cholangiocarcinoma. Furthermore, only a small subgroup of patients with PSC have dominant strictures (12/41 patients or 29% in this series). Single institution series invariably have small numbers of patients and it is therefore not possible to randomise treatment groups in a controlled study. Patients may also be receiving other therapies that could affect the outcome, and in this study all patients were continued on ursodeoxycholic acid and were given intravenous ciprofloxacin for a week following ERCP. Follow-up is usually over months and not several years. Despite these inherent problems which bedevil all PSC treatment studies, patients in this study seemed to benefit from endoscopic therapy, and the marked reduction in serum bilirubin levels was particularly encouraging. Serum bilirubin levels are probably the single most important predictor of outcome in PSC, and features in all prognostic models. However it has not been shown that interventions that reduce the serum bilirubin level have a favourable effect on prognosis, and three patients in this series required liver transplantation.

Of greater concern however is the unpredictable occurrence of cholangiocarcinoma which may arise in about 10–20% of PSC patients. Features suggestive of a complicating cholangiocarcinoma include rapid clinical deterioration in a patient with previously stable PSC, and progressive stricturing and duct dilatation on cholangiography. The presence of a high grade dominant stricture must always raise the suspicion of cholangiocarcinoma, and repeated biliary brushings may be required to detect malignant cells. Although no patient in this study developed cholangiocarcinoma, the follow-up may not have been long enough to detect this devastating complication. Unfortunately, there is currently no reliable way of predicting which patients with PSC will develop a cholangiocarcinoma. Screening tests (serum tumour marker CA 19–9 and CEA, and cytology of biliary brushings) are only helpful in a negative sense, i.e., patients found to have cholangiocarcinoma are excluded from transplant. There thus seems little justification in delaying liver transplantation in young symptomatic patients with advanced PSC and dominant strictures.

Liver transplantation for PSC has problems of its own, and there appears to be a higher complication rate following tranplantation for PSC than for other diseases causing end-stage liver failure, in particular a higher rate of biliary stricturing in the transplanted ducts [7]. Possible causes of the strictures include recurrent PSC, ischaemic bile duct injury, chronic ductopaenic rejection, and infectious cholangitis related to the Roux-en-Y anastomosis and immunosuppression. However, three year survival after liver transplantation for PSC is 85% at most centres [7]. Furthermore, it is the only treatment that removes the risk of cholangiocarcinoma in PSC (the risk for colonic carcinoma in patients with co-existing ulcerative colitis however remains). We would reserve the use of biliary dilatation for the small subgroup of patients with early PSC and a dominant stricture involving the distal bile duct. PSC is usually a diffuse disease of the biliary tree not amenable to local therapy and, in this enigmatic condition, it is particularly important to treat the patient and not the cholangiogram. Furthermore, endoscopic therapy for PSC may be technically very demanding, and should only be performed at a centre of endoscopic excellence with transplantation facilities, such as that of Professor Manns in Hanover.

### *References*

[1] Cameron, J. L.,, Gayler, B. W.,, Sanfrey, H.,, Milligan, F.,, Kaufman, S.,, Maddrey, W. C., and Herlong, H. F., (1984). Sclerosing cholangitis: anatomical distribution of obstructing lesions. *Annals of Surgery*, **200**, 54–60.

[2] Myburgh, J. A., (1994). Surgical biliary drainage in primary sclerosing cholangitis. *Archives of Surgery*, **129**, 1057–1062.

[3] Cameron, J. L.,, Pitt, H. A.,, Zinner, M .J.,, Herlong, H. F.,, Kaufman, S. L.,, Boitmott, J. K., and Coleman, J., (1988). Resection of hepatic duct bifurcation and transhepatic stenting for primary sclerosing cholangitis. *Annals of Surgery*, **207**, 614–622.

[4] Ismael, T.,, Angrisani, L.,, Powell, J. E.,, Hubscher, S.,, Buckels, J.,, Neuberger, J.,, Elias, E., and McMaster, P., (1991). Primary sclerosing cholangitis: surgical options, prognostic variables and outcomes. *British Journal of Surgery*, **78**, 564–567.

[5] Lemmer, E. R.,, Bornman, P. C.,, Krige, J. E. J.,, Wright, J. P.,, Beningfield, S.,, Jaskiewicz, K.,, Kirsch, R. E.,, Kahn, D.,, Terblanche, J., and Robson, S. C., (1994). Primary sclerosing cholangitis: requiem for biliary drainage procedures? *Archives of Surgery*, **129**, 723–728.

[6] Dickson, E. R.,, Murtaugh, P. A.,, Wiesner, R. H.,, Grambsch, P. M.,, Fleming, T. R.,, Ludwig, J.,, LaRusso, N. F.,, Malinchoc, M.,, Chapman, R. W., and Kaplan, M. M., (1992). Primary sclerosing cholangitis: refinement and validation of survival models. *Gastroenterology*, **103**, 1893–1901.

[7] Lee, Y.-M., and Kaplan, M. M., (1995). Primary sclerosing cholangitis. *New England Journal of Medicine*, **332**, 924–933.

Eric R Lemmer
MRC/UCT Liver Research Centre
Groote Schuur Hospital
Observatory, Cape 7925
South Africa

Phillipus C Bornman
Professor and Head
Surgical Gastroenterology E23
Groote Schuur Hospital
Observatory, Cape 7925
South Africa

